# Initiation and formation of stereocilia during the development of mouse cochlear hair cells

**DOI:** 10.3389/fcell.2026.1928332

**Published:** 2026-10-23

**Authors:** Suraj Ranganath Chakravarthy, Raj K. Ladher, Thomas S. van Zanten

**Affiliations:** 1 National Centre for Biological Sciences, Tata Institute for Fundamental Research, Bangalore, India; 2 Instituto de Nanociencia y Materiales de Aragón (INMA), CSIC-Universidad de Zaragoza and CIBER-BBN, Zaragoza, Spain; 3 Fundación Agencia Aragonesa Para la Investigación y el Desarrollo (ARAID), Zaragoza, Spain

**Keywords:** actin filament, hair cells, inner ear, kinocilia, microvilli, stereocilia

## Abstract

**Introduction:**

Stereocilia are apically located actin-protrusions of inner-ear hair cells that form graded staircase-like bundles essential for mechanosensation. Although stereocilia morphogenesis has been described in several species, quantitative analysis of this process in the mouse cochlea remains limited, and previous studies have relied largely on scanning electron microscopy.

**Methods:**

Here, we combined scanning electron microscopy and fluorescence microscopy to characterise hair cell apical surface development and stereocilia formation in the mouse organ of Corti from early specification through postnatal development.

**Results:**

Before hair cell differentiation, sensory progenitors exhibit a dense lawn of microvilli on their apical surface. Hair cell differentiation is accompanied by progressive remodeling of the apical surface, beginning with enrichment of junctional actin, followed by eccentric relocalisation of the kinocilium, microvillar blooming, and selective thickening of microvilli proximal to the kinocilium. These morphological changes are followed by further protrusion widening, row specialisation, and progressive microvillar resorption during postnatal bundle maturation. To quantitatively characterise the spatial organisation of F-actin during these transitions, we developed an unbiased normalized radial projection analysis (UN-RaPA), which maps relative actin filament distribution across individual cells while accounting for differences in cell shape, size and orientation. This approach enables comparison of actin signatures across developmental stages and cell types and can be extended to additional fluorescence channels. Using this multichannel capability, we mapped the spatial distributions of the actin-associated proteins Ezrin and Radixin onto developmental actin signatures, revealing distinct patterns associated with different stages of hair-cell and stereocilia development.

**Discussion and Conclusion:**

Together, our imaging and quantitative analysis framework defines developmental changes in the hair-cell apical surface and provides a quantitative approach for relating the spatial distribution of actin-associated proteins to morphological progression during stereocilia formation.

## 1 Introduction

An end-point of hair cell differentiation is the formation of stereocilia. These structures are the site of mechanotransduction in the inner ear, a function that is critically dependent on their structure (Furness et al., 1997). Each stereocilium is made up of bundles of highly crosslinked parallel actin filaments (Tilney et al., 1989; Velez-Ortega and Frolenkov, 2019). Stereocilia are arranged in graded rows that form a characteristic staircase pattern at the apical surface of the hair cell. These are next to an eccentrically positioned true cilium, the kinocilium. Together, this is called the hair bundle. Although stereocilia development has been well-characterised in avian systems (Tilney et al., 1992a; Tilney and DeRosier, 1986; Tilney et al., 1986), the exact ultrastructural changes marking stereocilia initiation in mammals remain incompletely resolved. Hair cells and their precursors are epithelial cells, and like most epithelial cells, hair cell precursors possess microvilli (mv) and a centrally located primary cilium. Microvilli, like stereocilia, are projections of actin filaments (Sharkova et al., 2023). In general, most epithelial cells, such as intestinal enterocytes, have microvilli of similar length (Crawley et al., 2014). In contrast, hair cells possess stereocilia that have varying length and width. Thus, even though stereocilia can be considered modified microvilli, it is the transition of microvilli to stereocilia that is a key event in the differentiation of a hair cell.

Stereocilia formation has been best characterised in the chicken basilar papilla (Tilney et al., 1992b), but has also been described in the mouse, rat and hamster cochlea (Kaltenbach et al., 1994; Lenoir et al., 1987; Li and Ruben, 1979; Zine and Romand, 1996). The earliest steps of differentiation follow the radial relocalisation of the kinocilium. At this stage, the microvilli of the nascent hair cell undergo a process often called “blooming” followed by their elongation. Subsequently, differential elongation establishes the staircase pattern. During differential elongation, supernumerary shorter rows of microvilli are resorbed, giving rise to the final, mature hair cell in the cochlea of most mammals. At each of these stages, actin-regulating proteins are likely to play a critical role in sculpting the microvilli into a staircase-patterned array of stereocilia (Avenarius et al., 2014). Indeed, mutations in proteins that bundle, cap, sever or anchor actin filaments, as well as myosin motor proteins that run on actin tracks, have been widely studied to understand the proximate causes for the establishment and maintenance of the stereociliary bundle (Park and Bird, 2023). However, their roles during early developmental processes, prior to any overt specialisation of stereocilia, remain poorly understood.

Mutations in actin-regulating proteins frequently show defects in other regions of the hair cell. As epithelial cells, hair cells have a circumferential junctional actin collar that maintains tissue integrity. In addition, stereocilia are anchored within the cuticular plate, a dense cortical actin network analogous to the terminal web (Pollock and McDermott, 2015). Perturbations in actin regulation may therefore impact multiple subcellular compartments, confounding interpretations on stereociliogenesis. A comprehensive, spatially resolved analysis of actin organisation throughout hair cell development is therefore needed to identify actin signatures associated with these morphological transitions. Moreover, a method that enables the distribution of actin-regulating proteins to be mapped onto these actin signatures would provide a means to assess their potential contribution to specific stages of hair-bundle morphogenesis.

Here, we characterise the development of microvilli and stereocilia bundles in the mouse cochlea from embryonic to postnatal stages. By quantifying their number and thickness, we identify developmental trends that extend previous observations and distinguish two broad phases of morphological change. The first is associated with kinocilium relocalisation, microvillar blooming, and selective thickening of microvilli, whereas the second is characterised by further widening of the protrusions, progressive row specialisation, and gradual microvillar resorption. Using a new analytical pipeline, we correlate morphological changes during stereocilia formation to patterns of actin organisation in the hair cell. We further use this framework to map the distribution of the actin-regulating proteins Ezrin and Radixin onto these developmental actin signatures, providing a quantitative approach to relate the spatial distribution of actin-regulatory proteins to specific stages of hair-bundle morphogenesis.

## 2 Methods

### 2.1 Animals

CD1 mice were used for all experiments. Gender of the animal was not determined prior to the experiment. Use of mice was carried out in accordance with institutional ethics guidelines.

### 2.2 Immunohistochemistry

Dissection and fixation of the inner ear in 4% paraformaldehyde (Sigma-Aldrich) overnight at 4° C was followed by the microdissection of the cochlea. For fluorescence microscopy analysis, samples from several protocols in different experiments were used. These differ by the type of permeabilisation and blocking but are based on previously used protocols (Hirono et al., 2004; Singh et al., 2022). Primary antibodies against Myosin VI (25-6791; *Proteus*), Pericentrin (PRB-432C; Covace), Radixin (R3653; Sigma-Aldrich) and Ezrin (E8897; Sigma-Aldrich) were all used at 1:200 dilution. Secondary antibodies (Thermo Fisher Scientific) goat anti-rabbit antibody conjugated with Alexa Fluor 546 was used at a 1: 500 dilution, and donkey anti-mouse with Alexa 594 and goat anti-rabbit with 647 were used at 1:200 dilution and incubated at room temperature for 1 h. Phalloidin was added with the secondary antibody and incubated for between 40 min and 1 h at room temperature. Both Alexa Fluor 488 and Alexa Fluor 555 conjugated Phalloidin (Thermo Fisher Scientific) were used. Slides were mounted with ProLong gold antifade (Thermo Fisher Scientific) and imaged using an Olympus FV3000 (60x oil immersion objective of 1.42 NA) or Zeiss LSM 980 Airyscan 2 (63x oil immersion objective of 1.4 NA). The imaging settings were typically kept constant for the same microscope but channel saturation was avoided by changing laser settings. For Airyscan image processing, the default option was used.

### 2.3 Scanning electron microscopy

Preparation of samples for scanning electron microscopy (SEM) were as previously described (Singh et al., 2022). Briefly, dissection of the inner ear in phosphate buffered saline and were then fixed in 2.5% glutaraldehyde (Sigma-Aldrich) in 0.1 M sodium cacodylate buffer pH 7.3 with 3 mM CaCl_2_ for at least 24 h. For P11 and P12 stages, inner ears were dissected after decalcification with 120 mM ethylenediaminetetraacetic acid (EDTA) (Montgomery and Cox, 2016), although in our modified protocol, this is in 0.1 M sodium cacodylate for 2 days. This was followed by microdissection of the Organ of Corti (OC) in 0.1 M sodium cacodylate buffer. The samples were then post-fixed in 1% osmium tetroxide (Sigma-Aldrich) for 1 h at room temperature. This is followed by treating the tissue with 0.5% thiocarbohydrazide (Alfa Aesar). Another round of osmium tetroxide and thiocarbohydrazide treatment was carried out. After the last osmium tetroxide fixation, the sample was dehydrated through an alcohol series and were then subjected to critical point dehydration (Leica EM CPD300). The samples were mounted with double sided carbon adhesive tape and sputter coated (Coating thickness ∼10 nm). Then the samples were imaged in Zeiss Merlin Compact VP. SE2 mode was used in imaging with a voltage of either 5 kV or 10 kV as indicated.

### 2.4 Image analysis

#### 2.4.1 Actin protrusion number and width analysis

IHC and OHC were identified morphologically from the SEM images. At E15.5, IHCs were readily identifiable based on their eccentric kinocilium and characteristic “bloomed” microvilli. The kinocilium of hair cells and the cilium of supporting cells were also distinguishable from microvilli by their greater apparent width. In contrast, OHCs were more difficult to identify unambiguously at this stage. Presumptive OHCs were identified as cells located lateral to the IHCs that exhibited a relatively elevated apical surface, with a kinocilium and microvilli bordered by slender microvilli extending from the adjacent supporting-cell surface. Postnatally, both IHCs and OHCs were readily identifiable, with the kinocilium typically positioned near the vertex and separated from the staircase-patterned stereociliary bundle. ImageJ was used to count microvilli/proto-stereocilia/stereocilia from identified HCs using the Cell-Counter plugin. We measured the widths of the actin protrusions from SEM images, of similar magnifications except for P11 and P12, by drawing a horizontal line across the shaft region of microvilli/proto-stereocilia/stereocilia and used the measure function (Hadi et al., 2020).

#### 2.4.2 Doming analysis

A cell-averaged slice through their apical cortex was generated by drawing a horizontal line through the center of the cells and applying the dynamic radial slice function. To quantify hair cell doming, a horizontal reference line was drawn immediately below the junctional contacts between hair cells and supporting cells. A perpendicular vertical line was then drawn from this reference line to the apical surface of the hair cell, or base of the stereocilia or microvilli. For flattened cells, the vertical line was drawn only to include the cell-cell junctions. The length of the vertical line was used as the measure of cell doming.

#### 2.4.3 Actin junction analysis

Actin junctions were analysed using the Python implementation of Tissue Analyser (Aigouy et al., 2016) from maximum-projected images (∼100 × 50 µm) of the basal region of the organ of Corti. Junctions were automatically identified, with minor manual corrections where required. Image and segmentation data were loaded into Napari and cells positive for myosin VI were annotated based on the myosin VI channel. Junctions were dilated from 1 to 3 pixels in width, and the mean actin intensity was extracted for each junction. To combine data from multiple sample preparations, junction intensities were normalised to the median intensity across all junctions, junctions directly adjacent to myosin VI-positive cells were then separated from those not adjacent to myosin VI-positive cells, and the resulting datasets were combined for analysis.

#### 2.4.4 Unbiased normalised radial projection analysis (UN-RaPA) for actin analysis

Multiple fluorescence microscopy images from a z-stack (300 nm slices) of a 100 µm region of the organ of Corti were collapsed in a single maximum projection image. Each pixel (80–100 nm) in this image contains information of relative F-actin density. From this image, cell types were identified, their cell boundaries were outlined, and the fonticulus was specified using Napari. These landmarks enabled the intensity values of each cell to be reprojected from Cartesian (x, y) into polar (theta, r) coordinates using a custom written program in Python. The Jupyter Notebook and instructions will be made freely available.

The reprojection involved extracting an intensity profile that started at the center-of-mass of each cell and extended to the cell boundary. Subsequently, this profile was rescaled from 0 to 1, being the center of mass and the cell-cell boundary, respectively. Intensity profiles were recorded each 4 degrees until the cell profile recording reached full circle, i.e., 360 degrees. The intensity profile that traverses the center of the fonticulus was defined as the 0 degrees angle and placed in the middle of the map. All other intensity profiles were reorganized accordingly, from −180 to 180 degrees. To be able to include the full cell boundary, each profile was extended by 40% from the cell boundary. The relative position of the F-actin-sparse fonticulus was determined by fitting Gaussian functions to the actin minimum associated with the fonticulus (r_f_) and the actin maximum associated with the cellular boundary (r_b_) in the 5-pixel wide averaged intensity profile at 0 degrees. The relative fonticulus position was subsequently determined as r_f_/r_b_.

UN-RaPA images from different developmental stages were used to assess the temporal progression of F-actin distribution in the three cell types: the Greater Epithelial Ridge (GER), Inner Hair Cells (IHC), and Outer Hair Cells (OHC). To enable comparison across cell types, sample preparations, and developmental stages, the reprojection images were normalized to the intrinsic F-actin intensity of the GER cell boundaries. For each image, the normalization factor was calculated from the mean intensity of the cell boundary (at r_b_) of GER cells. This allowed cells with different shapes and sizes to be combined into a single intensity heat map. The rescaled and reoriented heatmaps also enabled extraction of mean intensity from defined regions, such as the apical region of the cell. Because substantial deviations from the general elliptical cell shape introduce distortions that preclude direct comparison, UN-RaPA was applied to OHCs only up to P4-P5, after which their shape starts deviating substantially from an ellipse (Etournay et al., 2010).

#### 2.4.5 UN-RaPA for additional channels

To correct for spatial deviations between microscopy channels, multicolour 200 nm TetraSpack beads were imaged using identical microscope settings. Any translational shifts between channels were corrected based on the bead images. Cell landmarks identified in the actin channel were then used to reproject the corresponding maximum-projection images of the corrected additional channels.

For Pericentrin, intensity maps were analysed using the same procedure as for actin. The position of the Pericentrin peak in the 0-degree intensity profile was used to determine the radial offset between the Pericentrin signal and the F-actin-sparse fonticulus. The intensity profile at this radial position was then extracted, and fitting of a Gaussian function along the angular coordinate was used to determine the angular offset between the F-actin-sparse fonticulus and the Pericentrin peak. For comparison of Ezrin and Radixin intensities, each channel was normalised on a per-image basis using the mean cellular intensity of GER cells, following the same normalisation approach used for the F-actin polar heatmaps.

## 3 Results

### 3.1 Enrichment of actin filaments in hair cell junctions during early differentiation

Hair cells are first morphologically distinct, basally at E15.5, with clear differentiation evident at E18.5. This wave of maturation progresses to the apical ends of organ of Corti (Li and Ruben, 1979; Chen and Segil, 1999; Lim and Anniko, 1985). The expression of Atoh1, a key determinant of HC, follows a similar basal to apical gradient, although its expression is detected earlier in putative basal HC at E14.5, extending apically by E17.5 (Chen and Segil, 1999; Chen et al., 2002). To identify the earliest features of HC differentiation, we stained the organ of Corti from E14.5 to E16.5 mice with phalloidin (Figure 1A). We found that at E14.5, some cells at the base and the middle regions show enriched junctional actin. By E15.5, a greater number of cells with enriched junctional actin are observed in those regions, with some becoming detected in the apical regions as well. Cells displaying enriched junctional actin belts did not directly neighbour each other, suggesting that they were presumptive hair cells. These cells showed a spatial pattern typical of hair cell development, forming a discontinuous chain-like distribution along the basal-apical axis of the organ of Corti, with presumptive inner hair cells appearing before presumptive outer hair cells (Zine and Romand, 1996; Cohen et al., 2023). By E16.5, cells with enriched junctional actin were detected throughout the organ of Corti. In the basal regions, these cells additionally showed sub-cellular actin accumulation resembling nascent stereociliary bundles.

**FIGURE 1:**
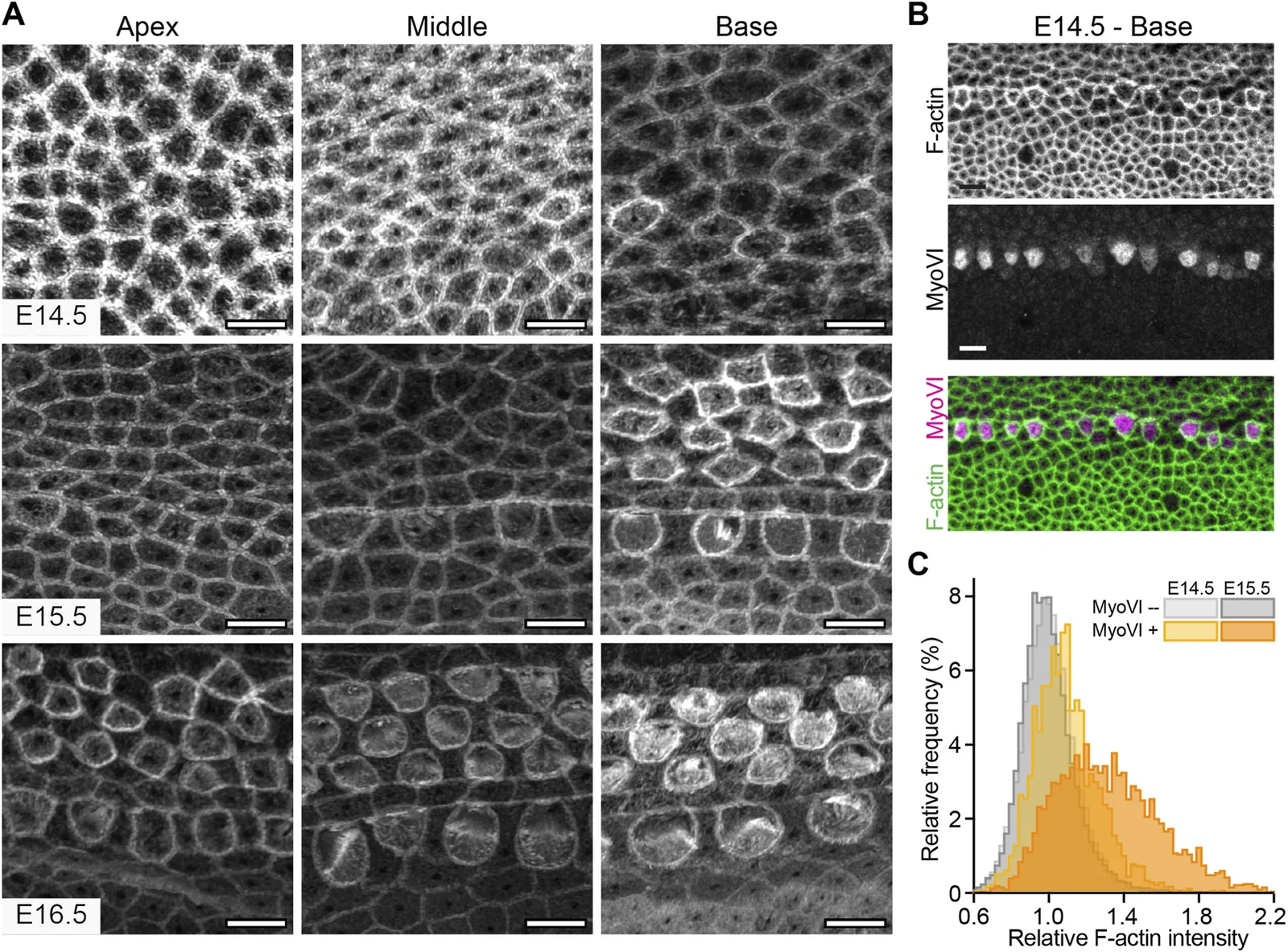
Differentiated hair cells have F-actin rich cell boundaries. **(A)** Super resolution airy scan images of phalloidin stained organ of Corti for the apical, middle, and basal region at E14.5, E15.5 and E16.5. Scale bars: 5 µm. **(B)** Two-colour image at E14.5 showing the spatial distribution of F-actin and MyoVI. Scale bar: 5 µm. **(C)** Relative F-actin intensity of cell junctions at E14.5 and E15.5 from all three regions of cochlea associated to cells lacking or expressing myosin VI. The data for E14.5 was obtained from 332 MyoVI+ and 13,415 MyoVI- cells (n = 5). The data for E15.5 was obtained from 672 MyoVI+ and 9117 MyoVI- cells (n = 7).

To quantify actin enrichment, we used Tissue Analyser to segment and annotate every cell junction within each field of view (Aigouy et al., 2016). To be able to confidently attribute actin enrichment to HCs we included a marker that is expressed in differentiating HCs: myosin VI (Figure 1B). Many of the cells that accumulate myosin VI also contain an actin belt that displayed an increased F-actin concentration compared to the cells that do not have myosin VI. The average intensity from each junction was measured and plotted as a frequency distribution for the basal regions of E14.5 and E15.5. We found that a population of junctions showed higher F-actin accumulation, and these were associated to cells expressing myosin VI. At the early stages, from E14.5 to E15.5, this population both increased in abundance and accumulated more F-actin. In contrast, cells that did not express myosin VI maintained the F-actin concentration in their cell boundary constant (Figure 1C). The delay in the appearance of cells with higher junctional F-actin intensities is apically correlated with the lag in the maturation of HCs in this domain. Moreover, the proportion and spatial pattern of cells showing higher F-actin mirrored the pattern of HC development (Figures 1A,C). This suggests that those cells with higher junctional F-actin are likely to be hair cells. We therefore propose that elevated junctional F-actin signature is a quantifiable early signature of the nascent HC differentiation and could be used to distinguish early differentiating hair cells from other cells in the organ of Corti.

### 3.2 Microvillar density demarcates early domains in the organ of corti

Although confocal microscopy showed differences in the levels of actin enrichment in clearly defined structures such as junctional cell boundaries, it is unable to resolve potential differences in features such as actin projections at the apical site of cells in the developing cochlea. To determine these, we used scanning electron microscopy. At E14.5, all cells showed microvilli (mv), defined as small actin protrusions of ∼75 nm in width. However, we observed significant regional differences in microvillar density between the abneural and neural regions of the organ of Corti (Figure 2A). Specifically, lateral, abneural regions of the organ of Corti showed reduced microvillar density (Figure 2B). In contrast, in medial, neural regions, mv density was higher (Figure 2B). Nonetheless, the putative sensory patch cells that are present in the neural region, did not show any heterogeneity with their neighbours, despite our observation of enriched actin in junctions of some of the cells.

**FIGURE 2:**
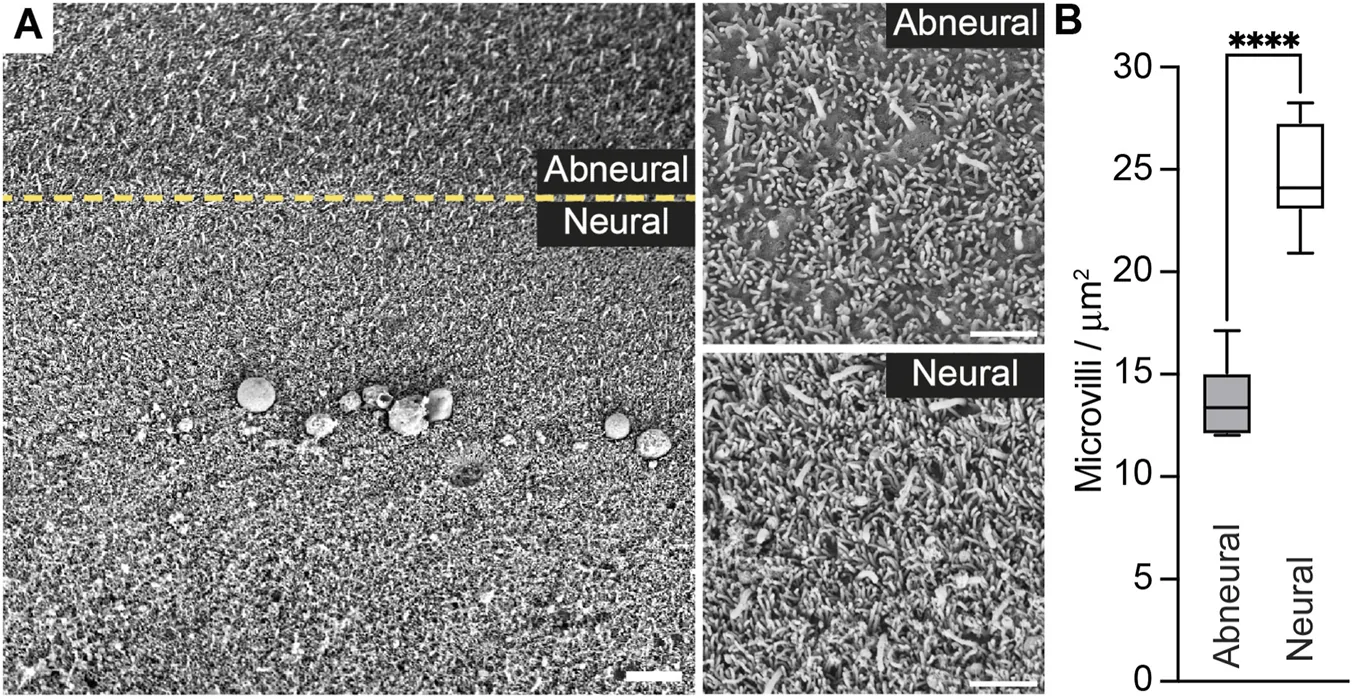
Increased density of microvilli in neural regions of the organ of Corti. **(A)** Large area scanning electron micrograph (SEM) of the organ of Corti at E14.5. Scale bar: 5 μm. Dashed line demarcates abneural from neural region of the sensory epithelium. Zoomed in regions of (A) display higher magnification SEM images showing microvilli at the surface of epithelial cells of the abneural region and the neural region. The latter is the presumptive hair cell region of the organ of Corti at E14.5. Scale bars: 2 μm. Images are from the end of the middle region close to the apical turn of the organ of Corti. **(B)** Quantification of the differences in microvilli coverage on abneural and neural regions, number of microvilli plotted per area (μm^2^). Microvilli were counted from 7 (abneural) and 8 (neural) 8 × 8 μm^2^ regions (n = 4). Bars represent mean ± std. ****P < 0.0001, ANOVA.

### 3.3 Kinocilia relocalisation correlates with increased HC microvilli number

Previous studies had observed that the first morphological indication of HC specialisation was an increased microvilli number when the kinocilia radially relocated, a process that has been termed blooming (Tilney et al., 1992a; Kaltenbach et al., 1994; Zine and Romand, 1996). Consistent with this, SEM imaging showed that putative inner hair cells (IHC) from the interface between the middle and base region at E15.5, and from the apical and middle region at E16.5 showed an increased number of mv (Figures 3A–C). To ask if we could identify signatures of blooming, we combined information from SEM with analysis from F-actin fluorescence confocal images. To account for cell-to-cell variability we reprojected the F-actin intensity values of each cell type into a normalized radial projection that reoriented and reshaped each cell into a matching and unbiased cartograph. To minimise any intensity variability between images that may have arisen due to differences in phalloidin staining or in imaging conditions, all intensity values were normalized to the average cell boundary intensity of the greater epithelial ridge (GER) cells. By determining the outline of each cell and pinpointing the F-actin sparse region (Figure 3D), Unbiased and Normalised Radial Projection Analysis (UN-RaPA) generates an intensity map for each cell type (Figure 3E). In the F-actin heat maps, the cell boundary is visible as a horizontal band of high intensity at r = 1 (Figure 3E; dashed lines). Intensity variation below this boundary displays the distribution of subcellular, including cuticular plate, apical actin content. GER cells, for example, display very little intensity variability within their apical cell cortex aside from a local decrease in F-actin content. This actin-sparse region of the cell apex corresponds to the fonticulus of an epithelial cell and is occupied by the cilium.

**FIGURE 3:**
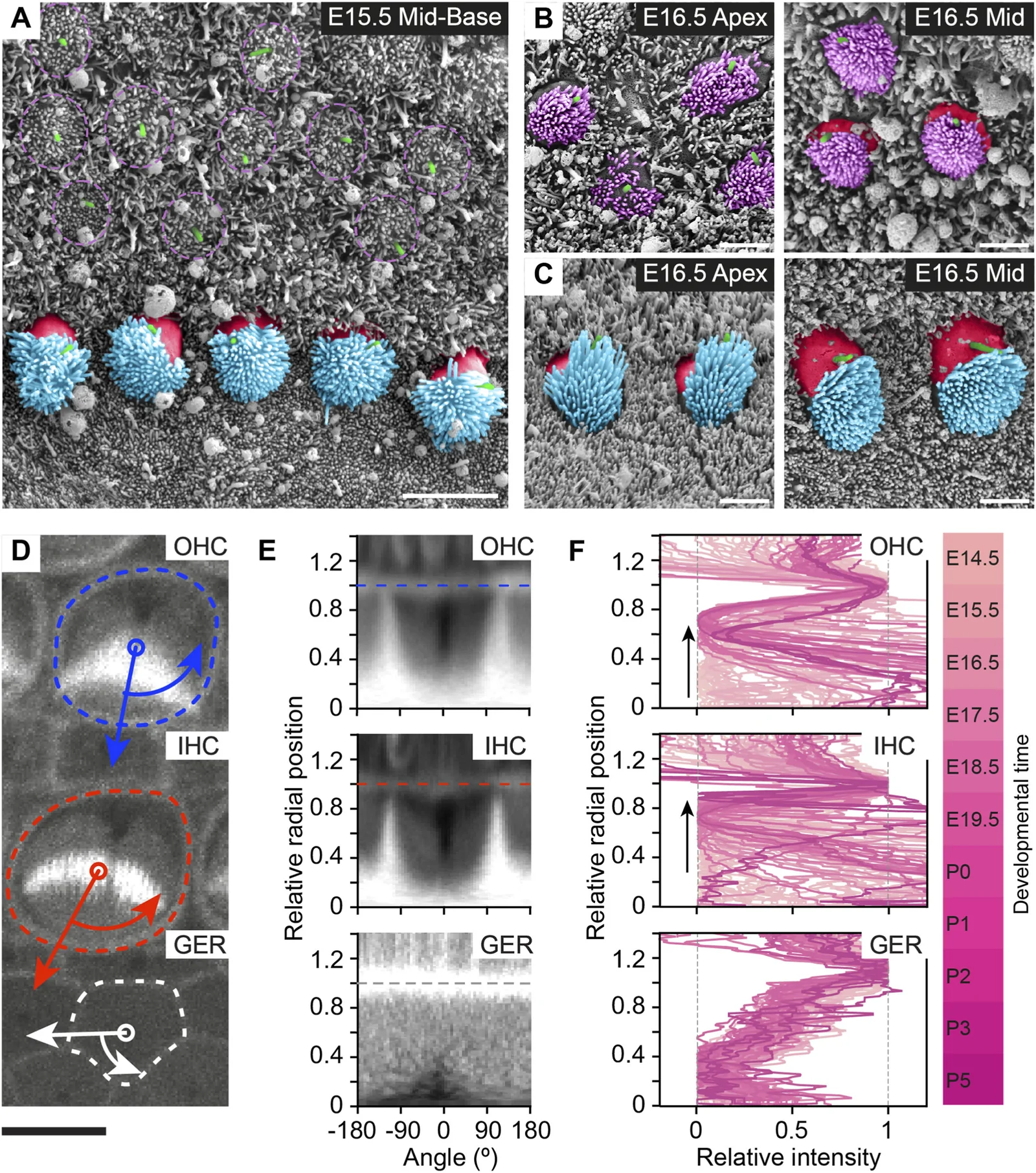
Developmental maturation of apical hair cell actin structures. **(A)** SEM image showing the surface of cells from the mid-base region of the organ of Corti at E15.5. Scale bar: 5 μm. **(B,C)** SEM images of OHCs **(B)** and IHCs **(C)** at E16.5 from the apical and middle region of the organ of Corti. Scale bars: 2 μm. False colouring of kinocilia (green colour), presumptive OHC (dotted purple circle), HCmv OHCs (purple), HCmv IHCs (cyan) and bare zone (red). Bilinear interpolation was used to false colour the SEM images using Adobe Photoshop. **(D)** Maximum projection confocal image of cells stained for F-actin (phalloidin) from the middle region of the organ of Corti at E19.5 showing an OHC, IHC and GER cell. For the UN-RaPA analysis, cells were identified, and their cell boundaries outlined (dashed line) together with their centroid (small circle). OHCs were identified in blue, IHC in red and GER cells in white. The curved arrow marks the direction and larger straight arrow marks extent of the radial slice outside the boundary of cells. Scale bar: 5 μm. **(E)** The UN-RaPA reprojection of the F-actin intensities into angle and relative radial position (r, with centre-of-mass and cell boundary representing 0 and 1, respectively) is displayed as a 2D heatmap for the three cell types: OHC (average of 36 cells), IHC (average of 12 cells), and GER (average of 12 cells). Dashed lines at r = 1 indicate the cell boundary for each of the three heat maps. **(F)** F-actin intensity traces obtained at different developmental timepoints from a single radial slice along the 0° angle of OHC, IHC, and GER cells. To facilitate visualization, each profile was normalized so its minimal cellular value is set to 0 and the intensity at the cell-boundary corresponds to 1. The arrows indicate the direction of the actin-sparse fonticulus positions as development progresses for OHC and IHC. The data was obtained from: E14.5 (n = 3), E15.5 (n = 5), E16.5 (n = 8), E17.5 (n = 3), E18.5 (n = 5), E19.5 (n = 3), P0 (n = 4), P1 (n = 4), P2 (n = 3), P3 (n = 1), and P5 (n = 1).

A summary of the intensity profiles extending from the center-of-mass until the cell-boundary at 0 degrees across developmental time, revealed limited profile change for GER cells (Figure 3F). In contrast, IHC or outer hair cell (OHC) cell populations displayed pronounced developmental shifts in their actin profiles. Dual color imaging with the centriole marker Pericentrin (Muhlhans et al., 2011) confirmed that the F-actin-sparse fonticulus coincided with the position of the cilium (Figure 4A). We therefore reasoned that the position of the fonticulus could be used as a proxy for the position of the cilium/kinocilium across developmental stages.

**FIGURE 4:**
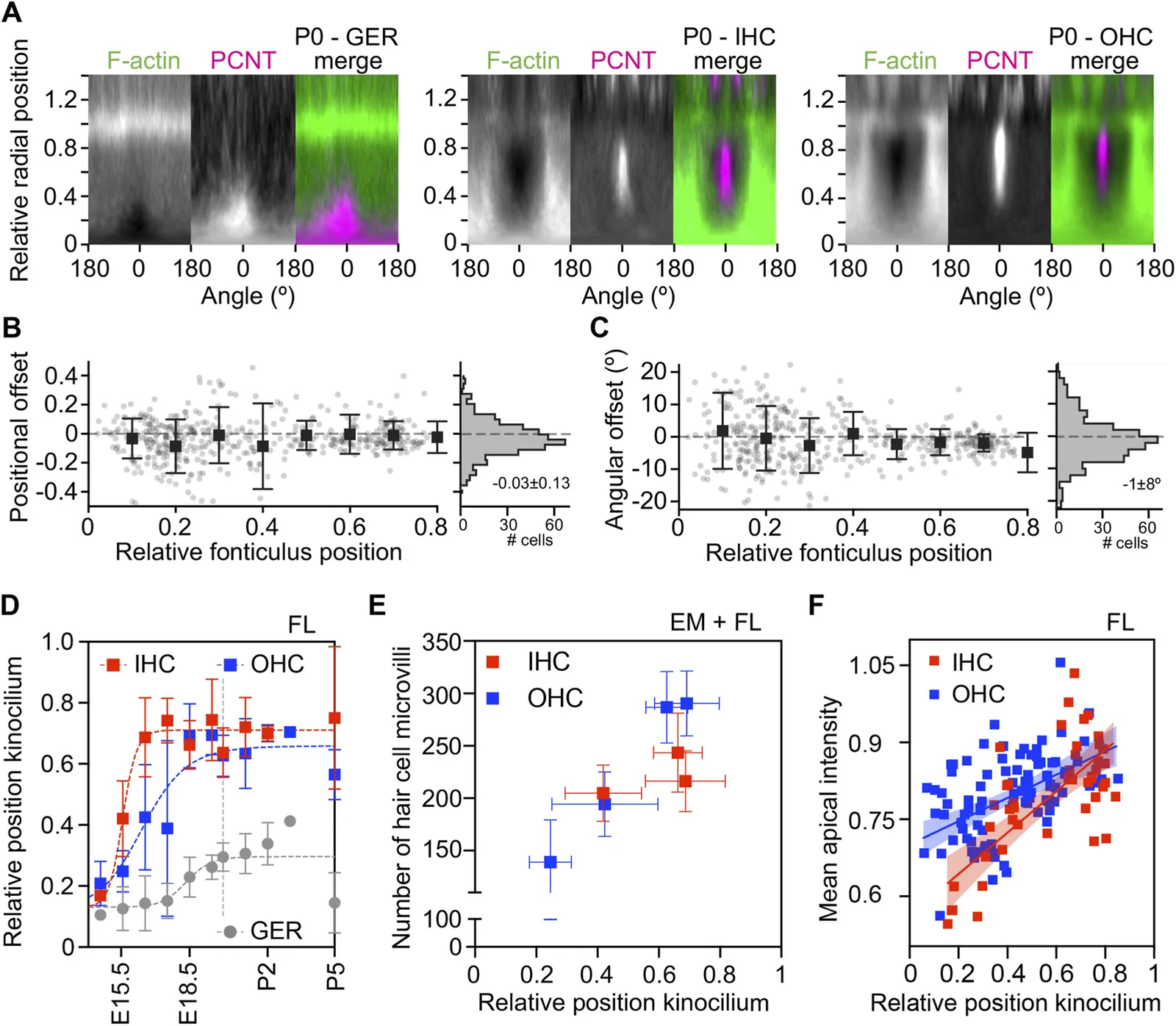
Relocalisation of kinocilia and initiation of Hair Cell microvilli (HCmv). **(A)** 2D heatmaps of dual labelled organ of Corti stained for F-actin and the centriole (Pericentrin, PCNT) showing the colocalisation between the F-actin-sparse fonticulus and the base of the kinocilium for the three cell types from the base region at P0. The 2D heatmaps were obtained by averaging over 43, 11 and 36 cells for OHC, IHC, and GER, respectively. **(B,C)** Scatter plots of the positional **(B)** and angular **(C)** offset between the F-actin-sparse fonticulus and the Pericentrin position as a function of the relative position of the fonticulus between cell centre-of-mass and boundary. Each dot corresponds to a single cell value, and the square points are the mean ± std of binned regions. Note that the positional offset is constant for all the possible fonticulus positions and the histogram centers at −0.03 ± 0.13. The variability of the angular offset decreases as the fonticulus moves to the cell boundary and has a mean of −1 ± 8°. Data was compiled from 464 cells imaged from the base region of the organ of Corti at E15.5 (n = 3) and P0 (n = 2). **(D)** Relative position, mean ± std, of the fonticuli over developmental timepoints tracks the relocalisation of kinocilium on hair cells and primary cilium on GER cells. The data was obtained from: E14.5 (30 GER, 30 IHC, 24 OHC), E15.5 (116 GER, 147 IHC, 297 OHC), E16.5 (314 GER, 383 IHC, 1221 OHC), E17.5 (66 GER, 85 IHC, 273 OHC), E18.5 (53 GER, 67 IHC, 225 OHC). E19.5 (67 GER, 78 IHC, 253 OHC), P0 (55 GER, 58 IHC, 200 OHC), P1 (109 GER, 158 IHC, 524 OHC), P2 (48 GER, 63 IHC, 193 OHC), P3 (14 GER, 16 IHC, 61 OHC), and P5 (33 GER, 48 IHC, 151 OHC). For associated n see caption of Figure 3. Each of the cell type datapoints have been fitted with a 4-parameter sigmoidal fit displayed in the graph as a dashed line. **(E)** Mean ± std of the number of HCmv from cells correlates to the relative kinocilia position obtained from cells at similar developmental stages. The HCmv were counted using scanning electron microscope and the position of kinocilia was assessed using UN-RaPA from the mid regions of the organ of Corti at E15.5-P0. Statistics of the fluorescence data was as above, SEM data came from: E15.5 (26 IHC, 18 OHC, n = 2), E16.5 (20 IHC, 23 OHC, n = 2), E18.5 (15 IHC, 16 OHC, n = 3), and P0 (21 OHC, n = 3). **(F)** Changes in the mean F-actin intensities on apical domain of HCs correlates with kinocilium relocalisation from the same cell (mid region of OC from E15.5 to E18.5). Note that each square is a single cell (47 IHC and 96 OHC). Solid lines indicate the fitted straight-line relationships obtained by nonlinear regression, with 95% confidence intervals shown as shaded regions, for IHCs (red) and OHCs (blue). Origin of the data sets is indicated in the figure panels as fluorescence microscopy (FL) and scanning electron microscopy (EM).

To assess potential positional or angular bias associated with using the fonticulus as a proxy, we collected dual-labeled cells from the basal region at E15.5 and P0, encompassing a broad range of fonticulus positions between the cell centre-of-mass and boundary (Figure 3F). We measured the positional offset between the fonticulus and Pericentrin across cells spanning this range of positions (Figure 4B). Importantly, neither the magnitude of the bias nor the variability of the measured offset changed with fonticulus position, with an overall normalised radial offset of −0.03 ± 0.13. Similarly, the angular orientation of cells determined using either the fonticulus or Pericentrin position showed a mean difference of −1 ± 8° (Figure 4C). Together, these measurements indicate that the fonticulus provides an accurate reporter of cilium/kinocilium position across developmental stages.

Having established the fonticulus as an accurate reporter of kinocilium position, we next quantified its developmental relocation relative to the cell boundary. At each developmental time-point, multiple profiles (each an average of >10 cells per z-stack) were fitted and combined to give an average ±standard deviation (std) position relative towards the cell boundary (Figure 4D). Using this analysis, we observed that relocalisation of the kinocilium was first detected for putative IHC of the mid-base region of the OC at E15.5 (Figure 4D). Kinocilial relocalisation for OHC was around a day later, at E16.5 in the middle region (Figure 4D). As radial migration of kinocilium has been associated with the increase in mv number in putative hair cells (Tilney et al., 1992a; Zine and Romand, 1996), we compared the measured number of mv obtained from SEM with the calculated kinocilium position obtained from UN-RaPA. Even though these data come from independently staged tissues that were measured with different imaging modalities, we found a positive correlation between mv numbers and kinocilial eccentricity for both IHC and OHC (Figure 4E).

We next asked whether signatures of microvilli blooming could be detected directly from F-actin fluorescence images. Although conventional confocal microscopy cannot resolve individual microvilli, the increased local density of microvilli should correspond to an increase in F-actin concentration and therefore increased intensity. The advantage is that kinocilium position and mv density can be extracted from a single cell compared to tissue-averaged values. Scatter plots for both IHC and OHC showed significant positive regression slopes between kinocilium displacement towards the cell boundary and the increase in F-actin density on the apical side of the hair cell (P < 0.0001, F-Test). The slopes differed significantly between IHCs and OHCs (P < 0.001, F-Test; Figure 4F). Together, both the SEM and confocal datasets provide clear signatures of mv blooming in developing mouse hair cells. Moreover, UN-RaPA data suggests that the temporal developmental program observed at the tissue level can be recapitulated in single cells and is different for IHC and OHC.

### 3.4 Hair cells dome during differentiation

As putative hair cells underwent differentiation, we observed doming, where the apex becomes elevated in comparison to the surrounding support cells (Figure 5A). This is particularly prominent from E16.5 to E18.5 in both IHC and OHC (Figures 5B,C). Quantifying Airyscan images along the x,z plane reveals that IHC doming can be first detected at E15.5 (Figure 5D). Here doming is defined as the height difference between the most apical F-actin cortex and the cell-cell junctional F-actin accumulation. At E15.5 this height difference is 1.7 ± 0.4 μm for IHC and reaches a maximum height of approximately 2.5 ± 0.2 μm at E19.5 and flattens as the hair cell further develops (Figure 5E). In OHC, a doming of 2.1 ± 0.3 μm is detected at E16.5 and appears to be oblate, with the region around the relocalised kinocilia being the highest point of the dome (Figures 5A,E). Due to doming, we were unable to accurately measure the length of the microvilli, although they appear to show some extent of differentiation lengthening.

**FIGURE 5:**
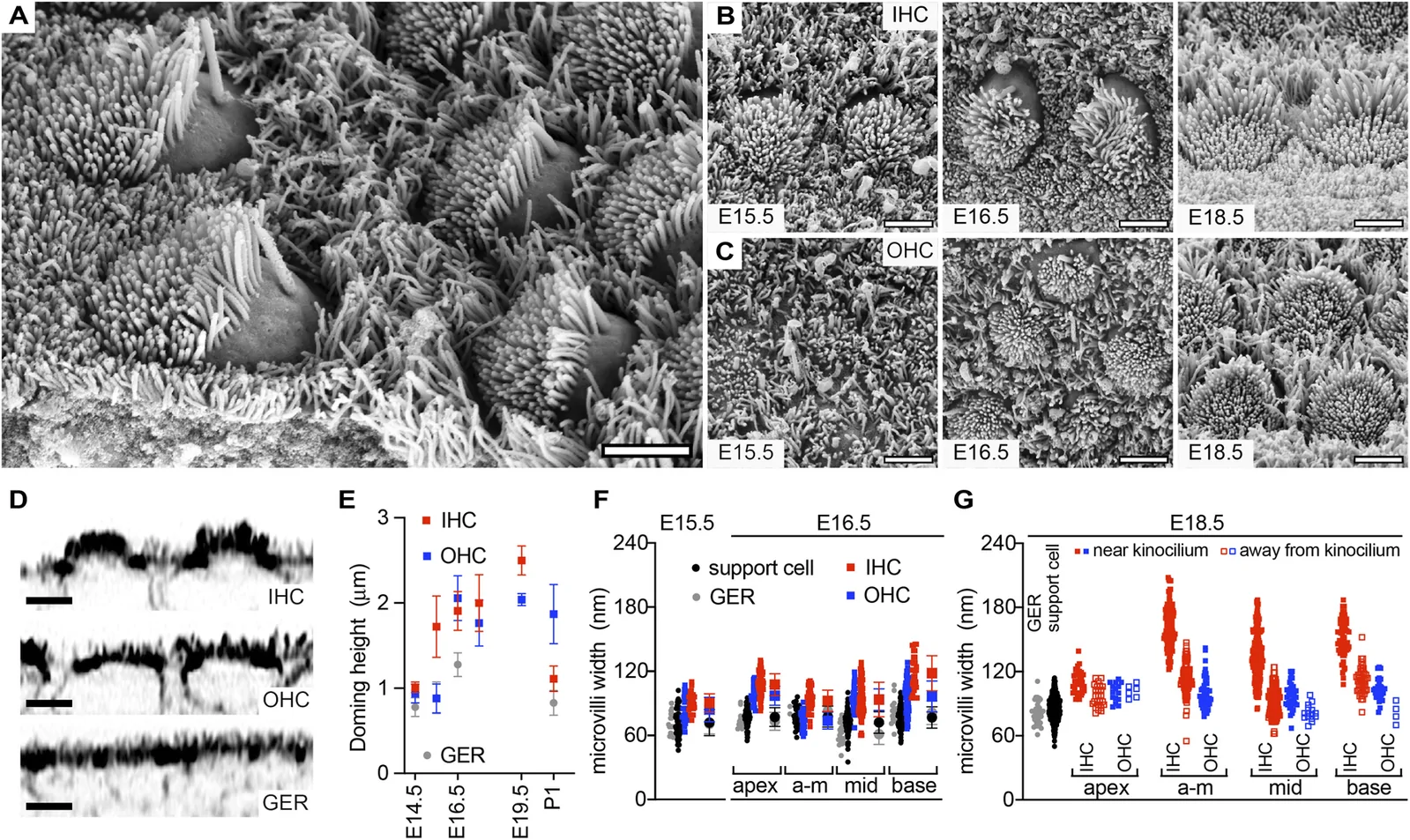
Sequence of events at embryonic stages leads to the specialisation of HCmv. **(A)** SEM (sideview) of the organ of Corti in basal turn at E18.5. Scale bar: 2 μm. SEM images of **(B)** IHCs and **(C)** OHCs at different developmental timepoints. All images are from the middle region of organ of Corti. Scale bars: 2 μm. **(D)** x-z slices of airyscan images of organ of Corti at E15.5 base stained with phalloidin 488. Doming was assessed in IHCs, OHCs and GER cells. Scale bars: 2 μm. **(E)** Quantification of doming, mean ± std, of apical surface in the IHC, OHC and GER cells from the basal region of organ of Corti across developmental timepoints from E14.5 to P1. Data of was from: E14.5 (14 GER, 17 IHC, 17 OHC), E15.5 (29 GER, 10 IHC, 40 OHC), E16.5 (13 GER, 7 IHC, 27 OHC), E17.5 (9 IHC, 28 OHC), E19.5 (3 IHC, 8 OHC), P1 (6 GER, 7 IHC, 22 OHC). **(F)** Graphs with widths of HCmv on IHCs (red) and OHCs (blue), microvilli on support cells (SCmv) (black) and microvilli on greater epithelial cell (GERmv) (grey) at the middle region of the organ of Corti at E15.5 and apical, interface of middle and apical, middle and basal regions of the organ of Corti at E16.5. Data came from: E15.5 - IHC (31 mv, 6 cells, n = 2), OHC (29 mv, 9 cells, n = 2), GER cells (11 mv, n = 1) and Support cell microvilli (69 mv, n = 2); E16.5 apex - IHC (60 mv, 11 cells, n = 2), OHC (27 mv, 7 cells, n = 1), GER cells (12 mv, n = 1) and support cell (80 mv, n = 2); E16.5 mid-apex - IHC (55 mv, 3 cells, n = 1), OHC (51 mv, 7 cells, n = 1), GER cells (2 mv, n = 1) and support cell (26 mv, n = 1); E16.5 mid - IHC (63 mv, 15 cells, n = 2), OHC (57 mv, 19 cells, n = 2), GER cells (20 mv, n = 1) and support cell (97 mv, n = 2); E16.5 base - IHC (31 mv, 8 cells, n = 2), OHC (81 mv, 16 cells, n = 2), GER cells (17 mv, n = 1) and support cell (94 mv, n = 2). **(G)** Graphs displaying the widths of microvilli on support cells (319 SCmv, n = 2: black), the widths of microvilli on greater epithelial cell (33 GERmv, n = 1: grey) and the widths of HCmv on IHCs (red) and OHCs (blue), separated in two pools based on their proximity to kinocilia (closed or open symbols for near or away from the kinocilium, respectively). The data was obtained from: E18.5 apex - IHC (36/25 mv, 5 cells, n = 1), and OHC (15/0 mv, 4 cells, n = 1); E18.5 mid-apex - IHC (136/106 mv, 7 cells, n = 1), and OHC (16/30 mv, 4 cells, n = 1); E18.5 mid - IHC (204/161 mv, 11 cells, n = 2), and OHC (37/15 mv, 7 cells, n = 2); E18.5 base - IHC (61/41 mv, 5 cells, n = 2), and OHC (30/4 mv, 3 cells, n = 1).

### 3.5 Specialisation of microvilli to stereocilia

Doming of hair cells precluded quantitation of microvilli length. Instead, we asked if quantifying the microvilli width could provide insights into the specialisation of stereocilia. To better understand the potential changes in mv thickness on hair cells, we used the developmental gradient of HC differentiation (Figures 1A, 5B,C). In the mid-apical region of OC at E15.5, we observe that the widths of mv on both presumptive IHCs and OHCs are equivalent (91 ± 9 nm and 85 ± 11 nm, respectively) and thicker (P < 5·10^−4^, ANOVA) than the microvilli on support cells (73 ± 12 nm) and GER cells (71 ± 9 nm) (Figure 5F). This is the first evidence of heterogeneity amongst the microvilli in the prosensory domain. IHCs in the mid-basal region bloom at E15.5 while blooming for OHC occurs around E16.5. Kinocilium relocalisation reaches its most eccentric position at E16.5 and E18.5 for IHC and OHC, respectively (Figure 4D). We therefore decided to measure HCmv width along the apical-base axis at E16.5 focusing on microvilli in proximity to the kinocilium. We hypothesised that at this time point, we would find the largest heterogeneity along the apical-basal axis. We find that HCmv on the IHCs progressively become wider (from 108 ± 10 nm to 119 ± 16 nm, P = 3·10^−4^, ANOVA) while the thickness of microvilli found on the supporting cells and GER cells remain constant (75 ± 10 nm and 72 ± 12 nm, respectively) (Figure 5F). In contrast, the HCmv on OHC do not display any change along the apical-basal axis of OC at E16.5, nor have they thickened significantly (91 ± 15 nm, P = 0.06, ANOVA). Altogether, the data suggests that heterogeneity amongst the microvilli of the sensory patch is first observed once hair cells attain their identity and the heterogeneity increases once the kinocilia has radially localised. We refer to these Hair Cell microvilli (HCmv) as actin protrusions that are thicker than 85 nm and arise as hair cells impart their characteristic bloom.

We next focused on HCmv width at E18.5, when kinocilium relocalisation had been completed (Figure 5G). At this time point we found a clear distinction between HCmv thickness depending on their proximity to the kinocilium (Figures 5B,C). We also observed that the HCmv width of IHCs were significantly larger in the basal region of the cochlea in comparison the ones found in the apical region of the cochlea (153 ± 21 nm versus 110 ± 10 nm) (Figure 5G). The population of HCmv from close to the kinocilia are wider (OHC P = 2·10^−4^, IHC P < 10^−4^, ANOVA) than those further away and the difference in thickness is larger for IHC (146 ± 24 nm versus 102 ± 17 nm) compared to OHC (100 ± 13 nm versus 82 ± 8 nm) (Figure 5G, excluding apex measurements). The entire HCmv population from IHC have increased significantly in width over the course of 3 days (E15.5-E18.5) whereas for the OHC only a subpopulation, those closest to the kinocilium, are thicker than microvilli from support cells of GER cells. This suggests that a subset of HCmv widen into an intermediate state, that we term proto-stereocilia, before they reach their final mature shape.

### 3.6 Early post-natal development of stereocilia

Proto-stereocilia were operationally defined as thicker HCmv located within the first four to five rows of HCmv, measured from the kinocilium side toward the cell interior. These protrusions subsequently widen and become organised into the characteristic row-wise staircase pattern during postnatal development (Figures 6A,B). Proto-stereocilia, closest to the kinocilium at E18.5 (134 ± 22 nm), continue to widen by 38 ± 1 nm per day and can be clearly designated as the first row by P2 and reach a width of 673 ± 38 nm at P12 (Figure 6C). At P2, the second and third row of stereocilia can also be distinguished, marking the transition from proto-stereocilia to stereocilia. Although starting out slightly wider at 282 ± 30 nm (compared to 232 ± 25 nm of the first row at P2), the second row of IHC follows a similar thickening rate as the first (39 ± 2 nm per day), reaching 661 ± 33 nm at P12. In contrast, the third row starts at 148 ± 22 nm, widening more slowly (8 ± 2 nm per day) to reach 223 ± 25 nm at P12. As with IHC, row identity for OHC (proto-)stereocilia becomes evident in the postnatal stage. At P2, rows 1, 2 and 3 attain widths of 152 ± 14 nm, 174 ± 24 nm, and 124 ± 10 nm, respectively (Figure 6D). The width increases slower than on IHC and is similar for all rows being 18 ± 2 nm, 11 ± 4 nm, and 12 ± 1 nm per day, for rows 1,2, and 3, respectively.

**FIGURE 6:**
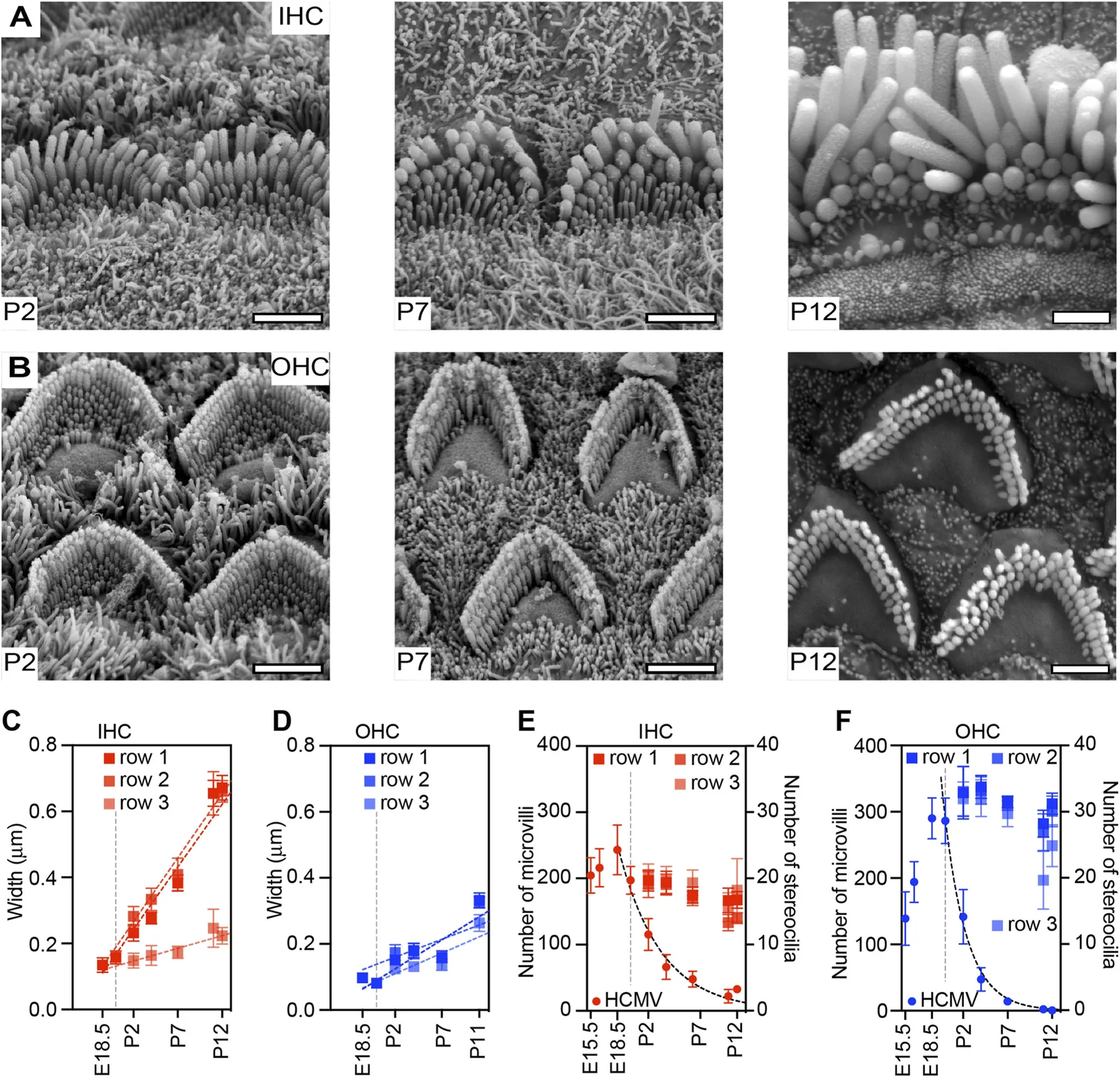
Development of Stereocilia. **(A,B)** Panel of SEM images at different developmental timepoints of IHCs **(A)** and OHCs **(B)**. Scale bars: 2 μm. All images are from the middle region of organ of Corti. **(C,D)** Widths of the three rows of stereocilia with row 1 closest to kinocilia at different developmental timepoints on IHCs **(C)** and OHCs **(D)**. Data on the width of row 1/2/3 were obtained from: E18.5 (204/0/0 on 11 IHC, n = 2; 37/0/0 on 9 OHC, n = 2), P0 (212/0/0 on 15 IHC, n = 3; 108/0/0 on 8 OHC, n = 2), P2 (79/52/81 on 15 IHC, n = 3; 55/45/73 on 22 OHC, n = 3), P4 (74/39/59 on 9 IHC, n = 3; 19/18/49 on 11 OHC, n = 2), P7 (43/23/39 on 8 IHC, n = 2; 26/26/59 on 15 OHC, n = 2), P11 (33/17/8 on 8 IHC, n = 2; 10/11/28 on 2 OHC, n = 1), and P12 (18/7/5 on 5 IHC, n = 1). The (proto-)stereocilia increase in width was fitted to a linear curve for all three rows, for both the IHC and OHC (dashed lines). **(E,F)** Plots displaying the mean ± std of the number of HCmv, and stereocilia in row1, row2 and row 3 on IHCs **(E)** and OHCs **(F)** as a function of developmental time. The data was obtained from: E15.5 (26 IHC, 18 OHC, n = 2), E16.5 (20 IHC, 23 OHC, n = 2), E18.5 (15 IHC, n = 3; 16 OHC, n = 2), P0 (19 IHC, 21 OHC, n = 3), P2 (13 IHC, 18 OHC, n = 3), P4 (15 IHC, n = 3; 13 OHC, n = 4), P7 (14 IHC, 16 OHC, n = 3), P11 (6 IHC, 46 OHC, n = 2), and P12 (4 IHC, 12 OHC, n = 1). The microvilli number decrease was fitted to a single exponential decay (dashed curve) that started at E18.5 or E19.5, for IHC or OHC, respectively.

Another feature of HC maturation is the resorption of excess HCmv (Kaltenbach et al., 1994). Soon after the appearance of proto-stereocilia, HCmv distant from the kinocilia begin to be resorbed. However, the rate of resorption is different for IHC and OHC. At E18.5, the HCmv population in the IHC starts falling at a constant exponential rate of 0.21 ± 0.02 days^-1^ (Figure 6E). Although the resorption of HCmv on OHC starts later, at around P0, resorption is faster than on IHC, at 0.40 ± 0.03 days^-1^ (Figure 6F). The difference between the two hair cell types is stark: the IHC loses about half of its HCmv plumage in 3.4 days while the OHC has lost it within 1.7 days. Moreover, the HCmv resorption on OHCs will continue until almost no HCmv are visible post P7. In contrast, around 33 ± 5 HCmv can still be found on IHC at P12.

At P0, the number of the hair cell microvilli that can be assigned as proto-stereocilia are 59 ± 9 for IHC and 106 ± 12 for OHC. By P2, the clear distinction in width between rows allowed stereocilia to be distinguished, and we counted a similar number of stereocilia in each row: 20 ± 2, 19 ± 2, and 20 ± 2 for IHC and 33 ± 4, 33 ± 4, and 32 ± 3 for OHCs counting for both rows 1, 2, and 3, respectively. Over time at P12 15%–25% of the stereocilia on both IHC and OHC have been resorbed (Figures 6E,F).

Altogether, the data suggests that as the stereocilia mature the hair cell actin machinery simultaneously operates two distinct processes. One designated to resorb and remove actin protrusions and a second tailored to widen and elongate a population of actin protrusions. We hypothesize that the transition of an actin protrusion towards one of the processes is associated with both its thickness and position, potentially reflecting a threshold-like behavior.

### 3.7 Ezrin to radixin switch coincides with stereocilia formation

ERM family proteins link the plasma membrane to the actin cytoskeleton and are known regulators of microvillar architecture (Bonilha et al., 2006; Saotome et al., 2004; Takeuchi et al., 1994). We next asked whether changes in ezrin and radixin localization accompany the transition from hair cell microvilli to stereocilia. Ezrin is required for the stabilization and growth of nascent microvilli in other epithelial systems, whereas radixin has been implicated in the maintenance of mature stereocilia structure in hair cells (Kitajiri et al., 2004; Pataky et al., 2004). We therefore used UN-RaPA to quantify the spatial distribution and relative abundance of ezrin and radixin during the developmental window spanning microvillar blooming, stereocilia commitment, and bundle maturation (E16.5–P4) (Figure 7).

**FIGURE 7:**
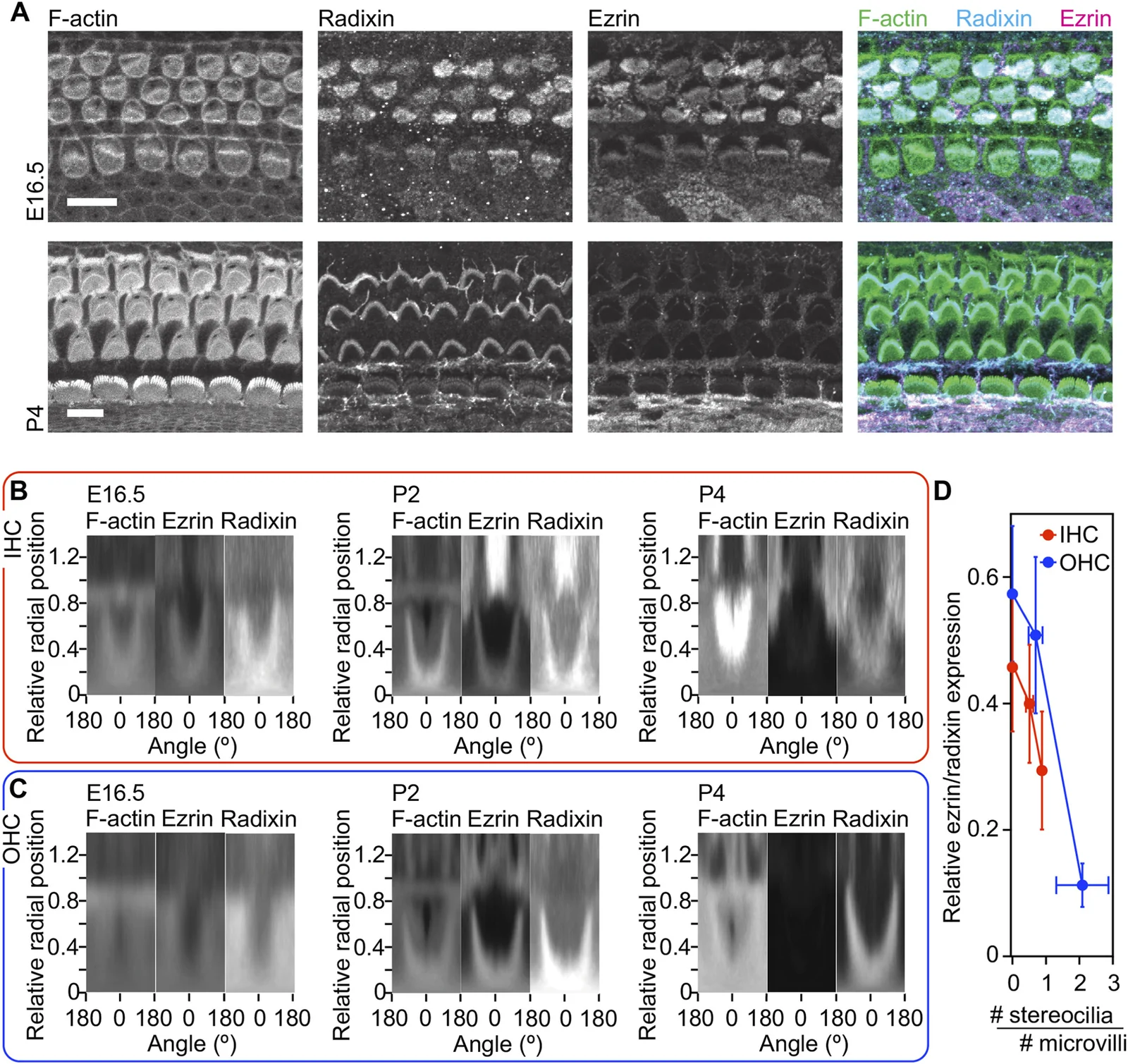
Expression and distribution of Ezrin and Radixin during Stereocilia development. **(A)** Three-colour images at E16.5 (top panel) and P4 (bottom panel) showing the spatial distribution of F-actin, Radixin and Ezrin at two developmental stages. **(B,C)** 2D UN-RaPA intensity maps of F-actin, ezrin and radixin displaying the relative distribution of the proteins at different developmental timepoints of IHCs **(B)** and OHCs **(C)**. The data was obtained from: E16.5 (47 IHC, n = 3; 144 OHC, n = 3), P2 (26 IHC, n = 2; 77 OHC, n = 2), and P4 (24 IHC, n = 2; 76 OHC, n = 2). The actin maps were normalized to the cortical F-actin intensity of GER cells and the ezrin and radixin intensities were normalized to the apical GER intensities from the following number of GER cells E16.5 (34 GER, n = 3), P2 (20 GER, n = 2), and P4 (14 GER, n = 2). **(D)** Relative expression level of Ezrin over Radixin at the three different developmental stages correlated to the relative number of stereocilia over HCmv. The ratio of stereocilia number versus microvilli number was derived from the data of Figures 6E,F.

At E16.5, both ezrin and radixin were broadly distributed across the apical surface of inner and outer hair cells and colocalized with regions of elevated F-actin intensity associated with the hair cell microvillar bloom (Figures 7A–C). At this stage, both proteins showed strong positive correlations with F-actin intensity, with Pearson’s correlation coefficients of 0.73 ± 0.12 and 0.57 ± 0.14 for ezrin and radixin, respectively, in inner hair cells, and 0.57 ± 0.18 and 0.49 ± 0.19 in outer hair cells. These data indicate that both ERM proteins spatially coincide with the developing hair bundle and is suggestive of their participation in shaping the hair bundle morphology.

By P2, as stereocilia rows became morphologically distinguishable, both ezrin and radixin became increasingly concentrated around the developing hair bundle. However, by P4, when microvillar resorption was nearly complete in outer hair cells, ezrin levels declined markedly, whereas radixin remained strongly associated with the mature stereociliary region (Figures 7A–C).

This transition was particularly pronounced in outer hair cells, where the temporal decline in ezrin closely paralleled the rapid loss of residual hair cell microvilli (Figures 6F, 7D). In contrast, inner hair cells retained a small but persistent population of apical microvilli-like protrusions, accompanied by sustained low-level ezrin enrichment at the flanks of the stereociliary bundle (Figures 6E, 7B).

Together, these findings reveal a developmentally regulated shift from an ezrin-enriched microvillar state to a radixin-dominant stereociliary state, coinciding with the progression from proto-stereocilia to the mature bundle architecture.

## 4 Discussion

Our study demarcates the earliest cytoskeletal and morphological changes associated with stereocilia initiation in the developing mouse cochlea. By combining scanning electron microscopy, fluorescence imaging, and quantitative actin mapping through UN-RaPA, we identify a temporally ordered sequence of events that begins with junctional F-actin enrichment, followed by kinociliary relocalisation, microvillar blooming, apical doming, proto-stereocilia formation, and postnatal bundle maturation. Together, these findings provide a quantitative developmental framework for the earliest stages of mammalian stereociliogenesis.

Classical work has defined milestones during the development of the hair bundle in inner ear hair cells (Tilney et al., 1992a; Tilney et al., 1992b; Kaltenbach et al., 1994; Zine and Romand, 1996). Prior to HC and SC differentiation, the epithelium consists of uniform cells with sparse microvilli. In the rat, Zine and Romand noted that prior to HC differentiation, two epithelial domains with different microvillar densities, a region abneurally consisted of microvillar sparse cells, and those located neurally were microvillar-rich (Zine and Romand, 1996). Our work in the mouse corroborates this: Two domains based on microvilli density can be observed until E14.5, before the onset of any overt HC or SC differentiation. The epithelium towards the neural edge, which will give rise to the sensory patch and GER, is microvillar-rich. More laterally, towards the abneural edge, the epithelium is microvillar-sparse. A central finding of this study is that elevated junctional F-actin represents one of the earliest detectable hallmarks of hair cell differentiation, preceding overt morphological specialization. This suggests that cytoskeletal remodeling at cell boundaries is an early event in fate acquisition and may contribute to the biomechanical changes required for subsequent apical remodeling.

The temporal relationship between junctional actin enrichment and the onset of apical doming raises the possibility that reinforcement of cortical tension is functionally linked to the emergence of the domed hair cell surface. Similar dome-like apical morphologies have been reported in other epithelial systems undergoing active surface remodeling. Doming has been observed in the endodermal epithelial cells of the rat visceral yolk sac where doming of cell surface correlates with an increase in the number and length of microvilli (Jollie, 1986). Similarly, uterine epithelial cells also exhibit doming with an increase in the microvilli density (Laird et al., 2020). In contrast, enterocytes doming has been suggested to be associated with decrease in adhesion molecules that normally support microvilli (Pinette et al., 2019). Indeed, in enterocytes, apical markers are decreased, not increased, because of doming. Here, the perturbation of the actin network in brush border enterocytes results in doming and a decrease in microvillar density (Postema et al., 2019). These observations suggest that doming is not necessarily a consequence of increased apical differentiation but may instead be associated with changes in the organisation or dynamics of the cortical actin network (Jollie, 1986; Laird et al., 2020; Pinette et al., 2019; Postema et al., 2019).

Our data support a two-stage model of stereocilia morphogenesis (Figure 8). In the first, embryonic phase, hair cells undergo kinociliary relocalisation, microvillar blooming, and selective thickening of microvilli proximal to the kinocilium, generating a proto-stereociliary state. In the second, postnatal phase, these proto-stereocilia undergo further widening and row-wise organization while excess microvilli are progressively resorbed. This model extends classical developmental descriptions from avian and rodent systems by providing a quantitative timeline of cytoskeletal transitions and by identifying intermediate structural states that precede mature bundle formation (Tilney et al., 1992a; Kaltenbach et al., 1994; Zine and Romand, 1996).

**FIGURE 8:**
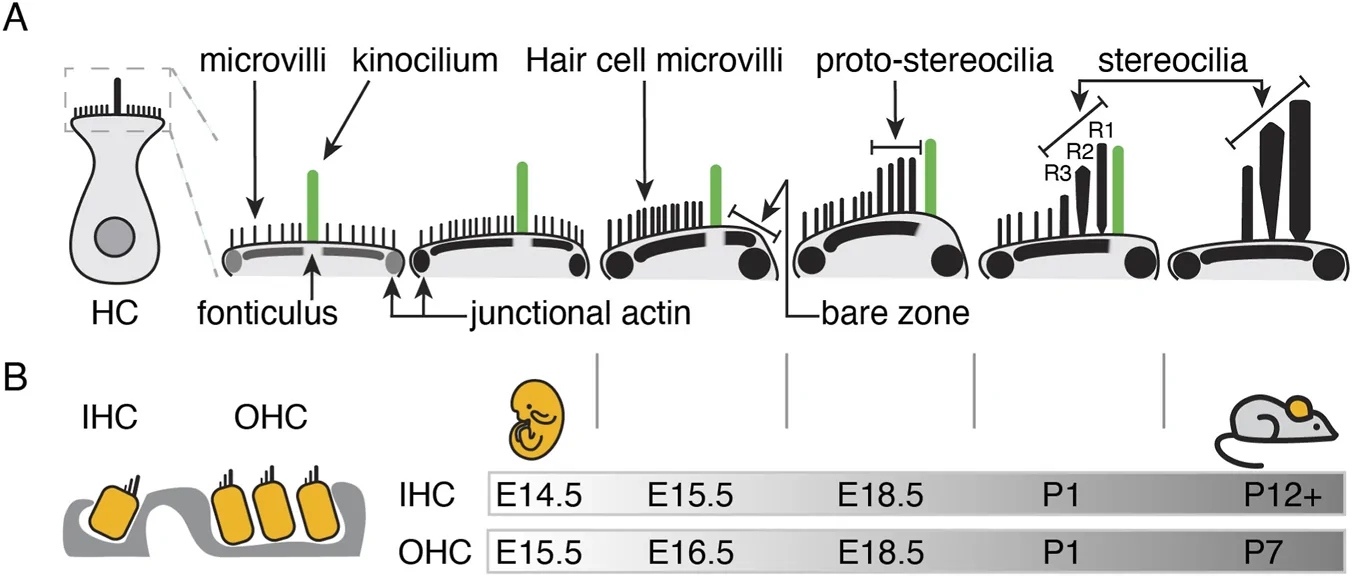
Schematic representation of hair cell apical surface development and stereocilia morphogenesis. **(A)** Schematic progression of the hair cell apical surface during hair-bundle development, illustrating the morphological sequence from hair cell microvilli (HCmv) through proto-stereocilia to the establishment and refinement of the stereocilia bundle. Early in development, presumptive hair cells acquire increased F-actin at their apical boundary. Following kinocilium relocalisation, differentiated hair cells develop a characteristic bloom of densely distributed HCmv. A subset of these protrusions subsequently specialises into the thicker proto-stereocilia. These thicker protrusions become organised into three rows (R1–R3) forming the developing stereocilia bundle, while the remaining HCmv are progressively resorbed during bundle refinement. Together, these morphological changes outline two broad phases of bundle development: an early phase characterised by HCmv blooming and selective protrusion thickening, followed by a later phase of bundle organisation, row specialisation and HCmv resorption. **(B)** Schematic overview of the developmental framework considered in this study, illustrating the sequential progression of hair-bundle development across developmental time, cochlear position (base, middle and apex), and hair cell type (IHC and OHC). IHC development precedes OHC development, and progression occurs with a basal-to-apical delay, with the middle and apical regions approximately 1 and 1.5 days behind the base, respectively.

The observation that microvillar thickening begins during embryogenesis but accelerates postnatally suggests that stereocilia maturation is not a single continuous process but instead proceeds through discrete developmental transitions (Krey et al., 2023). We propose that widening marks commitment of a subset of apical protrusions to a stereociliary fate, whereas thinner and more dynamic protrusions are selectively targeted for resorption. This interpretation is further supported by the differential kinetics of microvillar loss in inner versus outer hair cells, indicating cell-type-specific control of bundle refinement.

A particularly important molecular correlate of this transition is the shift from ezrin-rich to radixin-dominant apical architecture. Early in development, both proteins colocalize with the expanded apical F-actin-rich domain, consistent with a role in stabilizing the microvillar bloom. As development proceeds, ezrin declines in parallel with microvillar resorption, whereas radixin becomes concentrated in the mature stereociliary bundle. This developmental switch supports a model in which ezrin stabilizes an early, dynamic microvillar state, whereas radixin supports the maintenance of the mature mechanosensory bundle (Kitajiri et al., 2004; Pataky et al., 2004). The persistence of low-level ezrin signal in inner hair cells may reflect residual apical remodeling or maintenance of a small population of non-committed protrusions.

Beyond the biological findings, UN-RaPA represents an important methodological advancement. By normalizing actin intensities to the greater epithelial ridge and spatially reprojecting cells into a common coordinate system, this framework enables quantitative comparison of cytoskeletal organization across developmental stages, preparations, and experimental conditions. Importantly, the approach is readily extensible to mutant phenotyping and multiplex protein localization studies, providing a scalable framework for interrogating hair cell morphogenesis and disease-associated defects in bundle development.

Together, our findings establish a developmental and molecular framework for early stereocilia formation in the mouse cochlea and provide a quantitative platform for examining the relationship between cytoskeletal organisation and hair bundle morphogenesis.

## Data Availability

The raw data supporting the conclusions of this article will be made available by the authors, without undue reservation.
